# Influenza- and MCMV-induced memory CD8 T cells control respiratory vaccinia virus infection despite residence in distinct anatomical niches

**DOI:** 10.1038/s41385-020-00373-4

**Published:** 2021-01-21

**Authors:** Suzanne P. M. Welten, Josua Oderbolz, Vural Yilmaz, Susanna R. Bidgood, Victoria Gould, Jason Mercer, Roman Spörri, Annette Oxenius

**Affiliations:** 1 Institute of Microbiology, ETH Zürich, Vladimir-Prelog-Weg 4, 8093 Zürich, Switzerland; 2 MRC-Laboratory for Molecular Cell Biology, University College London, Gower Street, London, WC1E 6BT UK; 3 Institute of Microbiology and Infection, University of Birmingham, Edgbaston, Birmingham, B15 2TT UK

## Abstract

Induction of memory CD8 T cells residing in peripheral tissues is of interest for T cell-based vaccines as these cells are located at mucosal and barrier sites and can immediately exert effector functions, thus providing protection in case of local pathogen encounter. Different memory CD8 T cell subsets patrol peripheral tissues, but it is unclear which subset is superior in providing protection upon secondary infections. We used influenza virus to induce predominantly tissue resident memory T cells or cytomegalovirus to elicit a large pool of effector-like memory cells in the lungs and determined their early protective capacity and mechanism of reactivation. Both memory CD8 T cell pools have unique characteristics with respect to their phenotype, localization, and maintenance. However, these distinct features do not translate into different capacities to control a respiratory vaccinia virus challenge in an antigen-specific manner, although differential activation mechanisms are utilized. While influenza-induced memory CD8 T cells respond to antigen by local proliferation, MCMV-induced memory CD8 T cells relocate from the vasculature into the tissue in an antigen-independent and partially chemokine-driven manner. Together these results bear relevance for the development of vaccines aimed at eliciting a protective memory CD8 T cell pool at mucosal sites.

## Introduction

CD8 T cells are activated in an antigen-specific manner and have the ability to eliminate pathogens by producing effector cytokines and exerting cytotoxic functions. Upon viral infection, naive virus-specific CD8 T cells are activated, clonally expand, and give rise to a pool of effector cells capable of killing infected target cells. A small population of T cells persists as memory cells that have the capacity to respond and rapidly expand upon secondary antigen encounter. These long-lasting memory CD8 T cells are the basis for T cell-based vaccination approaches. Memory T cells form a heterogeneous population, where distinct subsets are defined based on differences in cell surface molecules, anatomical localization, proliferation capacity, effector functions and metabolism.^1^

Central memory T cells (T_CM_) express markers that permit lymph node homing and are therefore predominantly found in secondary lymphoid tissues, but these cells also recirculate. In addition, T_CM_ cells exhibit profound proliferative potential. Effector memory T cells (T_EM_) mainly recirculate and do not express lymphoid tissue homing molecules. One hallmark of these cells is their robust effector functions. Although both T_EM_ and T_CM_ recirculate in the vasculature, it is thought that the reactivation of T_CM_ cells is not immediate. Antigen first has to be transported to the lymphoid tissues where it is presented by professional antigen presenting cells to T cell zone-homing and resident T_CM_ cells. It is not entirely clear how T_EM_ cells are reactivated, but there is evidence that the size of the T_EM_ pool in peripheral tissues and blood is directly linked to its early protective capacity,^2–4^ indicating that these cells respond directly in the infected tissue. Circulatory memory T cells can also be divided into distinct subsets using the expression of the fractalkine receptor CX3CR1.^5,6^ Tissue resident memory (T_RM_) T cells are another subset of memory T cells, lodged in peripheral tissues, such as the lungs, salivary gland, gut, female reproductive tract, and the skin.^7–12^ In contrast to T_EM_ cells, T_RM_ cells are restricted from the circulation and are transcriptionally distinct from circulatory memory T cells.^13^ The initial signals that induce this phenotype depend on tissue-specific cues and include TGF-β, IL-15, and local antigen.^13^ T_RM_ cells rapidly exert their effector functions upon antigen encounter, leading to an anti-viral state in the tissue and to the attraction of other immune cells to the site of inflammation.^14,15^ Thus, memory T cells residing in peripheral tissues are poised for instant action and are located at barrier sites, which is where pathogens enter the body. Due to these characteristics, both T_RM_ and T_EM_ cells have gained interest in being exploited for vaccination purposes. However, it is not clear which T cell subset is superior in providing early protection upon secondary challenge in peripheral tissues.

In order to induce large numbers of effector-like T cells in peripheral tissues, cytomegalovirus (CMV)-based vectors are an interesting option. CMV infection induces an atypical CD8 T cell response, characterized by the accumulation of large numbers of effector-like T cells in the circulation, a process termed memory inflation.^4,16,17^ Maintenance of the inflationary T cell pool is dependent on antigen presentation by latently infected non-hematopoietic cells.^18^ Epitopes that induce inflationary T cell responses are processed by the constitutive proteasome, and this pathway can be utilized by inserting the epitope on the C-terminus of a gene of interest in the viral genome.^19,20^ In pre-clinical animal models, CMV-based vectors encoding foreign antigens derived from tumors or pathogens induced effector-like T cell responses specific for these inserted epitopes. These T cells provided protection from the relevant tumors or pathogens expressing these epitopes.^21–29^ The protective capacity correlated with the size of the inflationary T cell pool in the peripheral organ and blood.^2,3,29^

Here, we compared the local protective capacity of antigenic-specific CD8 T_RM_ cells (induced by influenza virus infection) and inflationary T cells (induced by MCMV infection) in lung tissue towards a secondary respiratory infection with vaccinia virus (VV) encoding the same antigen. We found that both influenza- and MCMV-induced memory T cells provided protection from a local VV challenge in an antigen-dependent fashion. However, the manner of the response was distinct. Whereas influenza-induced memory T cells increased the local pool of T cells by local proliferation and T cell recruitment, the MCMV-induced T cell pool migrated out of the vasculature into the lung parenchyma, which was partially driven by chemokines, enabling these cells to exert their effector functions at the site of infection.

## Results

### The phenotypic composition of the memory T cell pool in the lungs is virus dependent

**Fig. 1:**
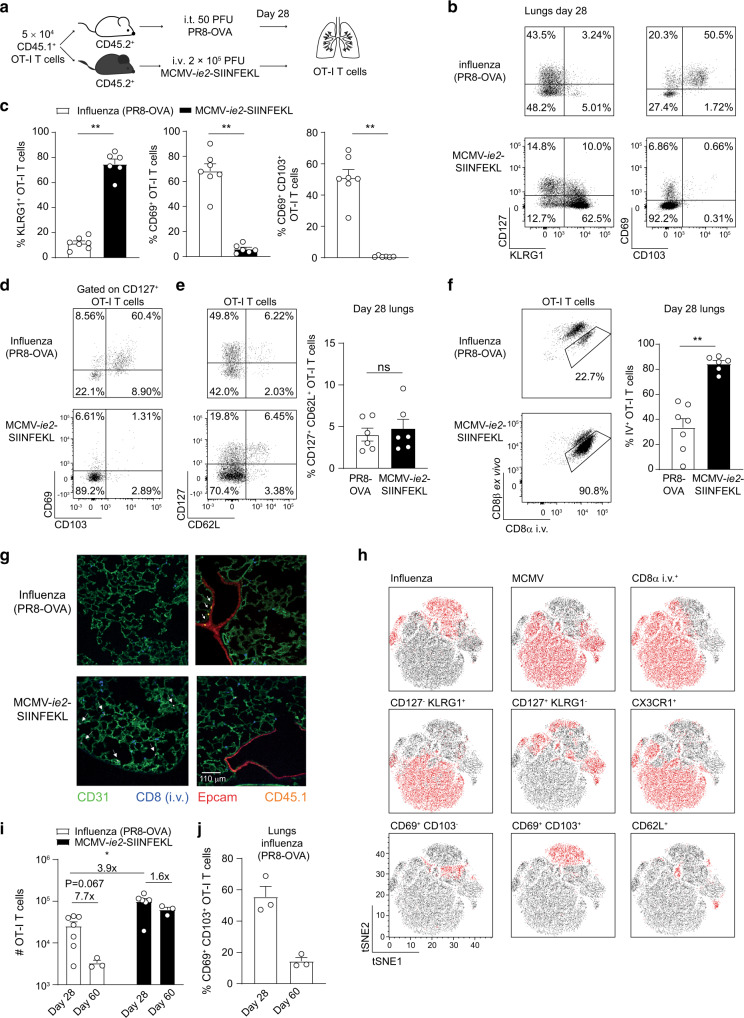
MCMV and influenza infection induce distinct memory CD8 T cell subsets in the lungs. **a** Experimental setup: 5 × 10^4^ CD45.1^+^ OT-I T cells were adoptively transferred into CD45.2^+^ hosts that were either infected intra-tracheally (i.t.) with 50 PFU influenza-expressing-OVA_257-264_ (PR8-OVA) or intravenously (i.v.) with 2 × 10^5^ PFU MCMV-expressing-OVA_257-264_ (MCMV-*ie2*-SIINFEKL). Twenty-eight days post infection, the phenotype of the OT-I T cells in the lungs was determined. **b** Representative flow cytometry plots show the cell surface expression of CD127 KLRG1 and of CD69 CD103 on OT-I T cells in the lungs. **c** Bar graphs show the percentage of OT-I T cells that expresses KLRG1, CD69 or CD69 CD103, indicated as mean + SEM. Data is pooled from two independent experiments (*n* = 6–7, each dot represents an individual mouse). **d** Flow cytometry plots show expression of CD69 CD103 on CD127^+^ OT-I T cells in the lungs 28 days post infection. **e** Flow cytometry plots show expression of CD127 CD62L on OT-I T cells in the lungs 28 days post infection. Bar graph shows the percentage of CD127^+^ CD62L^+^ OT-I T cells as mean + SEM. Data is pooled from two independent experiments (*n* = 6, each dot represents an individual mouse). **f** Mice received 5 µg fluorescently conjugated αCD8 antibody 3 min prior to euthanasia. Flow cytometry plots show in vivo CD8α labeling and ex vivo CD8β staining in the lungs, 28 days post-infection on OT-I T cells. Bar graphs indicate the percentage of OT-I T cells stained by the i.v. injected CD8 antibody as mean + SEM. Data is pooled from two independent experiments (*n* = 6–7, each dot represents an individual mouse). **g** Microscopy image shows the lung tissue 28 days after infection. Arrows indicate OT-I T cells. **h** tSNE clusters of CD45.1^+^ OT-I T cells isolated from the lungs of influenza- or MCMV-infected mice, based on expression of CD44, CD8α i.v., CD127, KLRG1, CX3CR1, CD69, CD103, and CD62L. Expression of each marker is color-coded with red = expressed and black = not expressed (*n* = 4 mice per group). **i** The total number of OT-I T cells recovered from the lungs is shown at day 28 and day 60 post-infection (*n* = 3–7). Fold difference is indicated. **j** The percentage of OT-I T cells in influenza virus infection that expresses CD69 CD103 is shown on day 28 and 60 post-infection, one experiment out of two is shown (*n* = 3), as mean + SEM, each dot represents an individual mouse. Two-sided Mann–Whitney test was used to determine statistical significance: *P < 0.05, **P < 0.01 and not significant (ns) *P* ≥ 0.05.

We established a model to compare the immediate protective capacity of distinct memory T cell pools in a peripheral tissue. We focused on the lungs as both T_RM_ cells and MCMV-induced inflationary T cells are established in this tissue.^30–32^ To compare memory T cells with the same specificity, naive CD45.1^+^ TCR transgenic OVA_257–264_/K^b^-specific (OT-I) T cells were adoptively transferred into CD45.2^+^ WT recipients before infection. Mice were then intra-tracheally (i.t.) infected with an influenza strain expressing the OVA_257–264_ epitope, PR8-OVA (Fig. 1a). A large pool of T_RM_ cells was induced, as the majority of the OT-I T cells in the lungs expressed the typical T_RM_ markers CD69 and CD103 four weeks post infection (Fig. 1b, c), but no sizeable KLRG1^+^ population was detected. To induce a large pool of effector-like cells in the lungs, mice were systemically infected with an MCMV-vector expressing the OVA_257–264_/K^b^ epitope under the control of the *ie2* promoter (MCMV-*ie2*-SIINFEKL), inducing an OVA_257–264_/K^b^-specific T cell response with inflationary T cell characteristics.^2,19^ The majority of the lung OT-I T cells expressed KLRG1, whereas hardly any expressed CD69 and CD103 (Fig. 1b, c), consistent with the effector-like phenotype of the MCMV-induced inflationary memory population. Similar results were found when endogenous OVA_257–264_/K^b^-specific CD8 T cells were analyzed instead of adoptively transferred TCR-transgenic OT-I cells (Supplementary Fig. 1A, B). In both influenza-induced and MCMV-induced memory CD8 T cells, a proportion expressed interleukin 7 receptor subunit alpha (IL7Rα, CD127). For influenza-induced OT-I T cells, this marker was mostly expressed by the T_RM_ CD69^+^ CD103^+^ cells (Fig. 1d). In both settings, few OT-I T cells co-expressed CD127 and CD62L, representing T_CM_ cells (Fig. 1e). Although administration of MCMV via the respiratory route was reported to induce MCMV-specific T_RM_ cells in the lungs,^33–35^ in our experimental setup both i.t. and systemic administration of MCMV predominantly induced an effector-like population of MCMV-specific CD8 T cells, and i.t. administration of the virus only slightly increased the proportion of T_RM_ cells (Supplementary Fig. 2A, B). This discrepancy might be explained by differences in genetics of the used mouse strains. We therefore henceforth used i.t. influenza virus infection to induce a CD8 T_RM_ population and systemic MCMV infection to raise an effector-like CD8 memory population.

T_RM_ cells are restricted from the circulation and located deep within tissues. To discriminate between cells within the tissue and those within/close to the vasculature, a fluorescently conjugated αCD8 antibody was i.v. injected into mice shortly before euthanasia. As expected, the majority of influenza-induced memory OT-I T cells was not labeled by this procedure, whereas MCMV-induced OT-I T cells stained mostly positive for the i.v. injected antibody, indicating that these cells are within or close to the vasculature (Fig. 1f). Similar results were observed for endogenous OVA_257–264_/K^b^-specific CD8 T cells (Supplementary Fig. 1C). The distinct localizations of the memory OT-I T cells were confirmed by microscopy. Influenza-induced memory OT-I T cells were located in close proximity to Epcam^+^ epithelial cells lining the lung airways and were not labeled by the i.v. injected αCD8 antibody. In contrast, MCMV-induced OT-I T cells were found in close proximity to CD31^+^ vascular endothelial cells and labeled by the short intravascular exposure to the αCD8 antibody (Fig. 1g).

In addition, we evaluated the phenotype of MCMV- and influenza-specific OT-I memory T cells using T-distributed stochastic neighbor embedding (t-SNE) analysis and observed heterogeneity between the different memory T cell pools (Fig. 1h). Besides phenotypical differences, the magnitude of the lung memory OT-I T cell pool was also different, as four times more OT-I T cells were detected in the MCMV setting (Fig. 1i). The number of MCMV-specific OT-I T cells in the lungs did not change drastically over a time period of 60 days. In contrast, 60 days post influenza virus infection, a ~6-fold reduction in the number of OT-I T cells in the lungs was observed (Fig. 1i). The T_RM_ cells in particular were declining in the lung tissue, as the percentage of CD69^+^ CD103^+^ OT-I T cells had dropped considerably at 60 days post influenza virus infection (Fig. 1j). This is in agreement with previous studies showing that T_RM_ cells in the lung are not steadily maintained.^36,37^ These findings were confirmed for endogenous OVA_257–264_-specific CD8 T cells (Supplementary Fig. 1D). The decrease in OT-I T cells in influenza infection was not observed in the spleen (Supplementary Fig. 1E). Combined, these results show that influenza virus and MCMV infection induce memory CD8 T cell populations in the lungs with distinct features in terms of their phenotype, localization, and maintenance.

### T cells induced by MCMV or influenza virus exhibit comparable protective capacity

**Fig. 2:**
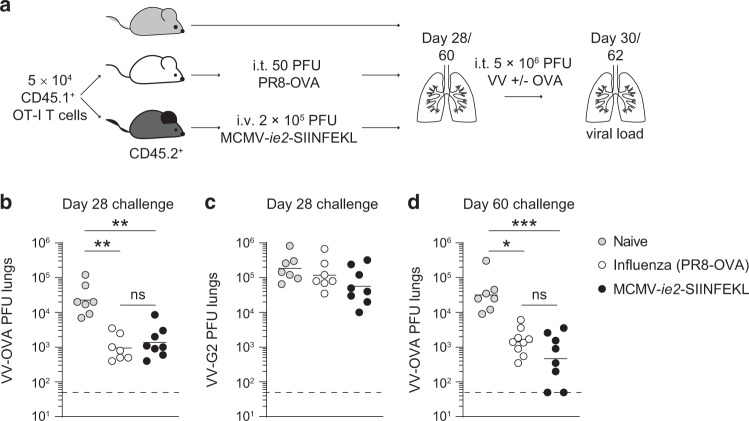
MCMV- and influenza-induced memory T cells protect from a local vaccinia virus challenge in the lungs in an antigen-specific manner. **a** Experimental setup: mice with memory T cells in the lungs induced by influenza virus or MCMV (as described in Fig. 1a) were challenged i.t. on day 28 (**b**, **c**), or day 60 (**d**) with 5 × 10^6^ PFU vaccinia virus expressing OVA (**b**, **d**) or with 3 × 10^5^ PFU VV-G2 (vaccinia virus that does not express OVA) (**c**). Two days post-challenge the viral load was determined in the lungs. All data is pooled from two independent experiments (*n* = 3–5). Each dot represents an individual mouse, geometric mean and limit of detection are indicated. Statistical significance was determined using one-way ANOVA with Kruskal–Wallis post hoc test to correct for multiple comparisons: *P < 0.05, **P < 0.01, ***P < 0.001 and not significant (ns) *P* ≥ 0.05.

To address the question whether the protective capacity of the distinct memory T cell compositions in the lungs differ, we performed a challenge infection with a recombinant VV expressing the shared OVA_257–264_ antigen (VV-OVA). Specifically, influenza- or MCMV-experienced mice were exposed to a local (i.t.) VV-OVA infection and the viral load in the lungs was determined 2 days later (Fig. 2a). A group of naive mice was included that harbored no pulmonary memory OVA_257–264_/K^b^-specific T cells before the VV-OVA challenge. Both groups of memory mice showed enhanced control of a local respiratory VV challenge as compared to naive mice (Fig. 2b). However, no differences in the viral load were observed between influenza- or MCMV-experienced mice. This result indicates that the distinct memory CD8 T cell compositions protect equally well from VV-OVA infection, irrespective if the majority of the memory T cell pool harbored T_RM_ or effector-like cells. Examining the protective capacity of endogenous OVA_257–264_/K^b^-specific memory CD8 T cells revealed comparable results (Supplementary Fig. 3). The protective effect of the memory OT-I T cells was antigen dependent, as challenge with a VV that did not express the cognate shared antigen was not controlled above naive mice (Fig. 2c). Since the number of T_RM_ cells in the lungs of influenza virus infected mice declined in time, mice were challenged with VV-OVA at a later time point to determine if the protective capacity would also diminish compared to MCMV-primed mice. However, despite the reduction in T_RM_ cells, mice that had memory T cells in the lungs, irrespective of whether they were induced by influenza virus or MCMV, showed enhanced control of VV-OVA challenge (Fig. 2d). These results show that mice with memory CD8 T cells in the lung tissue exhibit enhanced control from a local VV rechallenge, irrespective of the phenotypic memory T cell composition.

### T cells induced by influenza virus or MCMV infection produce effector cytokines

**Fig. 3:**
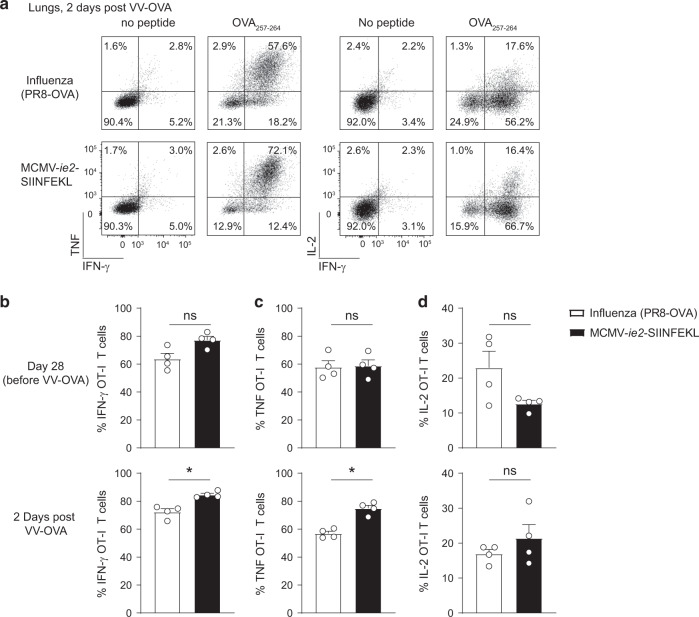
Memory T cells induced by MCMV or influenza virus produce effector cytokines. Cytokine production was determined in OT-I T cells from the lungs of MCMV-infected or influenza virus-infected mice, before (28 days post primary infection, top row) and 2 days post (bottom row) challenge with 5 × 10^6^ PFU VV-OVA. **a** Representative plots show intracellular production of IFN-γ versus TNF (left) and IL-2 (right). **b**–**d** Bar graphs show the percentage of OT-I T cells that produces IFN-γ (**b**), TNF (**c**), or IL-2 (**d**). All bar graphs represent mean + SEM and each dot represents an individual mouse. One experiment out of two independent experiments is shown. Mann–Whitney test was used to determine significance: *P < 0.05 (*n* = 4) and not significant (ns) *P* ≥ 0.05.

Memory CD8 T cells in the lungs provided protection from local challenge in an antigen-dependent manner. As the memory OT-I cells induced by influenza virus or MCMV infection had different characteristics, we next addressed whether the mechanism of responding to antigenic challenge would differ. IFN-γ and TNF produced by CD8 T cells are critical for the clearance of a respiratory VV infection.^38,39^ We examined the quality of the responding memory T cells by examining cytokine production before (28 days post primary infection) and 2 days post-secondary challenge with VV-OVA. OT-I T cells produced IFN-γ, TNF, and IL-2 (Fig. 3a), independent of which virus was initially used for the establishment of the memory pool. Also, after VV-OVA challenge, the majority of cells in both settings was able to produce these effector cytokines (Fig. 3b–d). To conclude, memory CD8 T cell populations induced in the lungs by either MCMV or influenza virus are both capable of producing effector cytokines upon secondary exposure to cognate antigen.

### The lung OT-I T cell pool increases in size upon antigenic rechallenge

**Fig. 4:**
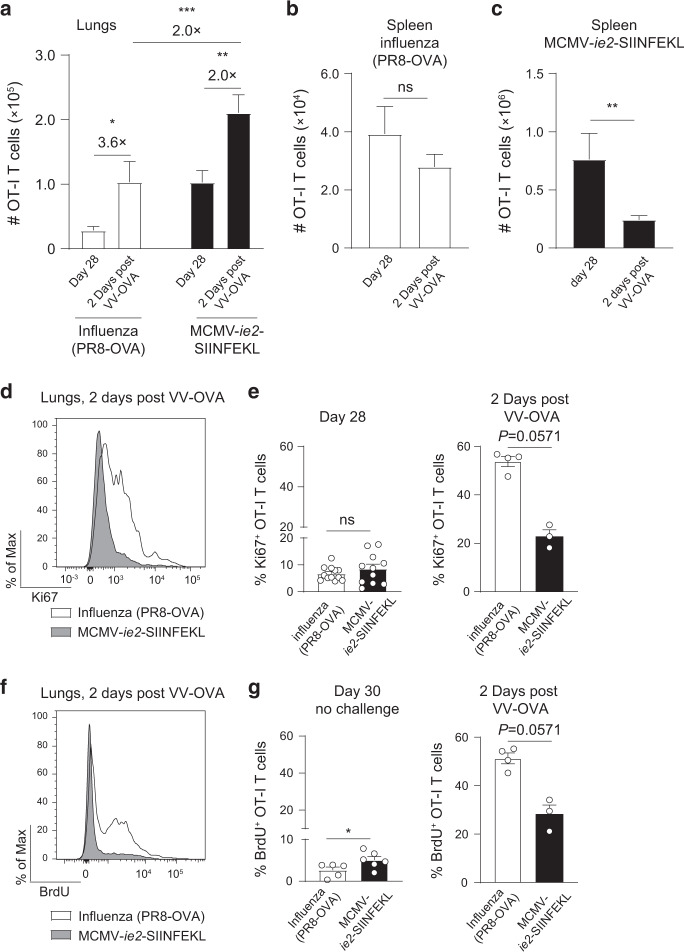
Memory T cells in the lung expand upon local rechallenge. Mice with memory OT-I T cells induced by influenza virus (PR8-OVA) or MCMV-*ie2-*SIINFEKL (as described in Fig. 1A) were i.t. challenged on day 28 with 5 × 10^6^ PFU VV-OVA. **a** The number of OT-I T cells in the lungs was determined 28 days post-priming and 2 days post-secondary challenge with VV-OVA (*n* = 18–31, pooled from eight independent experiments). **b** Number of OT-I T cells in the spleen 28 days post influenza virus (PR8-OVA) infection and 2 days post-secondary challenge with VV-OVA (*n* = 20–26, pooled from eight independent experiments). **c** Number of splenic OT-I T cells 28 days post MCMV-*ie2*-SIINFEKL infection and 2 days post-secondary challenge with VV-OVA (*n* = 16–31, pooled from eight independent experiments). **d** Histogram shows the expression of Ki67 in lung OT-I T cells 2 days post VV-OVA infection. **e** Bar graphs show the percentage of Ki67^+^ within OT-I T cells on day 28 post-primary infection (left) (*n* = 11–12, pooled from five independent experiments) or 2 days post-secondary infection with VV-OVA (right) (*n* = 3–4, one experiment out of eight is shown). **f** Mice received BrdU in the drinking water during the VV-OVA rechallenge period. Histogram shows BrdU incorporation in OT-I T cells in the lungs. **g** Bar graphs show the percentage of OT-I T cells that incorporated BrdU in mice from day 28 to day 30 (left) (*n* = 5–6, pooled from two independent experiments) and in mice that received a VV-OVA challenge on day 28 (right) (*n* = 3–4, one experiment out of 4 is shown). All bar graphs represent mean + SEM, each dot represents an individual mouse. Two-sided Mann–Whitney test was used to determine statistical significance: *P < 0.05, **P < 0.01, ***P < 0.001 and not significant (ns) *P* ≥ 0.05.

Infection with MCMV-*ie2-*SIINFEKL induced a memory OT-I T cell pool in the lungs that was higher in magnitude as compared to the T_RM_ pool induced upon influenza (PR8-OVA) virus infection (Fig. 1i). Next, we addressed the size of the OT-I T cell pool 2 days post challenge with VV-OVA. In both the MCMV and influenza virus setting, the OT-I memory T cell population increased in magnitude after local antigen re-exposure upon VV-OVA infection (Fig. 4a). Although the overall expansion of the OT-I T cell pool in the lung was more pronounced in the influenza virus setting (3.6-fold versus 2-fold), the population was still 2-fold larger in the MCMV setting. Also, the number of cytokine-producing cells was larger in the MCMV setting (Supplementary Fig. 4A–C). In contrast to the lung, no changes in the magnitude of the OT-I T cell pool were detected in the influenza virus setting in the spleen (Fig. 4b). Strikingly, in the MCMV setting, splenic OT-I T cells were reduced upon i.t. VV-OVA infection (Fig. 4c), likely indicating that splenic OT-I cells migrated and accumulated in the lung tissue. To corroborate the importance of T cell recruitment for the expansion of the lung localized OT-I T cell pool during VV-OVA challenge, mice were treated with FTY720 which inhibits S1P-S1PR-axis-mediated lymphocyte egress from lymphoid organs. In both the influenza- and MCMV-setting, the lung localized OT-I T cell pool was reduced compared to the untreated group, indicating that T cell recruitment was contributing to the expansion of the lung-localized OT-I T cell pool (Supplementary Fig. 4D).

Another explanation for the increase in magnitude of the OT-I T cells localized in the lung could be local proliferation. We therefore determined Ki67 expression in OT-I T cells to identify cycling cells. A higher percentage of OT-I T cells in the lungs expressed Ki67 in the influenza virus setting after local VV-OVA challenge  (Fig. 4d, e), although Ki67 was also expressed in a small population of MCMV-induced OT-I T cells. To corroborate these findings, mice were administered with BrdU in the drinking water during the recall response to determine active DNA synthesis and proliferation. Relatively more OT-I T cells induced by influenza virus infection incorporated BrdU compared to the MCMV setting upon VV-OVA challenge (Fig. 4f, g). In summary, these data show that lung-localized memory T cells induced by influenza virus or MCMV infection expand upon local viral rechallenge. However, a higher proliferative capacity was found in memory T cells induced by influenza virus infection. Together, these results suggest that the increased number of OVA-specific CD8 T cells in the MCMV-setting after local VV-OVA challenge might be primarily due to relocation of circulating cells to the lung tissue.

### Memory CD8 T cells induced by MCMV virus infection relocate from the vasculature upon local inflammation

**Fig. 5:**
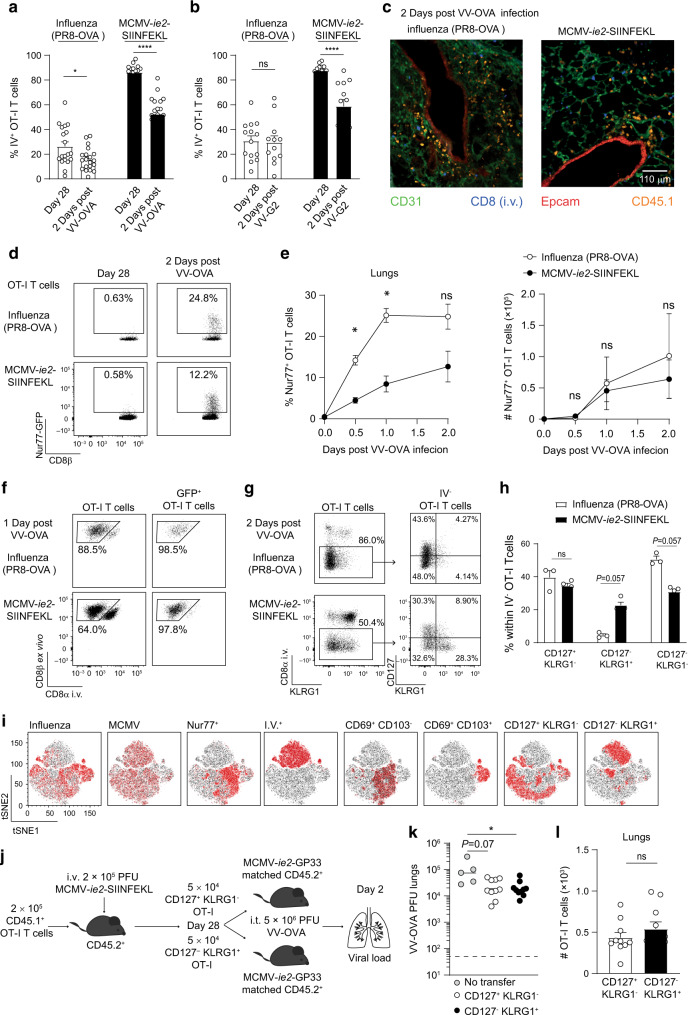
MCMV-induced memory OT-I T cells relocate in the lungs upon antigenic challenge. **a** Mice that either have memory OT-I T cells in the lungs induced by MCMV-*ie2-*SIINFEKL or influenza virus (PR8-OVA) (as described in Fig. 1a) were locally challenged on day 28 with 5 × 10^6^ PFU VV-OVA. 2 days post-secondary challenge, the percentage of OT-I T cells that was in the vasculature was determined by i.v. injection of 5 μg fluorescently conjugated αCD8 antibody 3 min prior to euthanasia (*n* = 14–19, pooled from five independent experiments). **b** Mice that either had memory OT-I T cells in the lungs induced by MCMV-*ie2-*SIINFEKL or influenza virus (PR8-OVA) (as described in Fig. 1a) were locally challenged on day 28 with 3 × 10^5^ PFU VV-G2 (a vaccinia virus that does not express the SIINFEKL epitope). 2 days post-secondary challenge, the percentage of OT-I T cells that was in the vasculature was determined by i.v. injection of 5 μg fluorescently conjugated αCD8 antibody 3 min prior to euthanasia (*n* = 12–14), pooled from four independent experiments). **c** Microscopy image shows the lung tissue 2 days post-infection with VV-OVA. **d** 5 × 10^4^ CD45.1^+^ Nur77-GFP OT-I T cells were adoptively transferred into host mice that were subsequently i.t. infected with 50 PFU PR8-OVA or i.v. with 2 × 10^5^ PFU MCMV-*ie2*-SIINFEKL. After 28 days, mice were i.t. challenged with 5 × 10^6^ PFU VV-OVA. Flow cytometry plots show Nur77 expression on OT-I T cells in the lungs before rechallenge (left) and 2 days post VV-OVA infection (right). **e** The percentage (left) and total number (right) of OT-I T cells expressing GFP (Nur77) is shown in the lungs, after VV-OVA infection as mean ± SEM (*n* = 2–4, one out of two experiments is shown). **f** Flow cytometry plots show in vivo CD8α labeling and ex vivo CD8β staining on all OT-I T cells (left) or on GFP^+^ OT-I T cells (right) one day post VV-OVA infection in the lungs. **g** Flow cytometry plots show in vivo CD8α labeling versus KLRG1 expression on OT-I T cells, gated on CD45.1^+^ in the lungs, 2 days post VV-OVA infection (left), and CD127 KLRG1 staining on the IV^−^ fraction of the OT-I T cells (right). **h** Percentage of cells expressing CD127 and/or KLRG1 within the IV^−^ fraction is shown (*n* = 3–4, one representative out of 3 independent experiments is shown). **i** tSNE clusters of CD45.1^+^ OT-I T cells in the lungs 2 days post VV-OVA infection, based on expression of Nur77, CD8α i.v., CD127, KLRG1, CD44, CD69, and CD103. Expression of each marker is color-coded with red = expressed and black = not expressed (*n* = 3 mice per group). **j** Experimental setup: 2 × 10^5^ OT-I T cells were adoptively transferred into naive hosts that were subsequently infected with 2 × 10^5^ PFU MCMV-*ie2*-SIINFEKL. After 4 weeks, CD127^+^ KLRG1^−^ and CD127^-^ KLRG1^+^ were sorted from the spleen. 5 × 10^4^ cells of each population were separately transferred into MCMV-*ie2*-GP33 infection-matched recipients, that were subsequently challenged with 5 × 10^6^ PFU VV-OVA. Two days later the viral load was determined in the lungs. **k** The VV-OVA viral load in the lungs is shown, pooled data from two independent experiments is shown (*n* = 5–10). Geometric mean and limit of detection are indicated. **l** The total number of OT-I T cells in the lungs is shown (*n* = 10, pooled from two independent experiments). All bar graphs show mean + SEM, each dot represents an individual mouse. Two-sided Mann–Whitney test (**a** and **j**) or two-way ANOVA followed by Sidak’s multiple comparisons test (**e**) or Kruskal–Wallis test with Dunn’s post hoc test (**i**) was used to determine statistical significance: *P < 0.05, ****P < 0.0001 and not significant (ns) *P* ≥ 0.05.

Next, we addressed if the localization of the memory OT-I T cells in the lungs would change upon challenge with a viral infection sharing the cognate antigen. As shown before, the majority of the MCMV-induced OT-I cells in the lungs was close to or within the vasculature (Figs. 1d, 5a). However, after local VV-OVA administration, a large fraction of the OT-I T cells migrated out of the vasculature deeper into the lung parenchyma, as determined by the absence of binding by an i.v. injected, fluorescently conjugated αCD8 antibody (Fig. 5a). Extravasation of OT-I T cells was also observed upon local challenge with a VV not expressing the OVA antigen (VV-G2), indicating that antigen was not a prerequisite for the relocation to occur (Fig. 5b). Moreover, when VV-OVA was administrated i.p. instead of i.t., OVA_257–264_/K^b^-specific CD8 T cells in the lung did not relocate deeper into the tissue. However, in the ovaries, which is the organ where VV replicates upon an i.p. infection, OVA_257–264_/K^b^-specific CD8 T cells extravasated (Supplementary Fig. 5A). Combined these results indicate that local inflammation is required for the extravasation of MCMV-induced memory T cells into the site of the infected tissue.

For memory T cells induced by influenza virus infection and responding to a VV-OVA challenge, we also observed a decrease in the percentage of cells that was stained by the i.v.-injected antibody (i.e. identifying cells within or close to the vasculature). This effect was antigen dependent, as it was not observed upon challenge with VV-G2 (Fig. 5b), likely reflecting an increase of cells in the lung tissue due to local proliferation of the lung localized cells.^40,41^ Microscopy analysis of lung sections showed relocalization of the MCMV-induced OT-I T cells from CD31^+^ endothelial cells closer to the Epcam^+^ epithelial cells lining the airways. These cells were not labeled by the i.v. injected fluorescently conjugated αCD8 antibody, confirming the flow cytometry data (Fig. 5c). Also, influenza virus-induced memory CD8 T_RM_ cells relocated from their original location near Epcam^+^ epithelial cells to more dispersed sites within the lung tissue.

To examine where in the lung tissue OT-I cells encountered antigen, we used OT-I T cells carrying a Nur77-green fluorescent protein (GFP) transgene and thereby reporting T cell receptor (TCR) triggering^42^ to generate lung memory OT-I T cells. A larger percentage of the influenza virus-induced memory T cells responded to antigen compared to MCMV-induced T cells, judged by a higher percentage of GFP^+^ OT-I T cells at all time points of analysis (Fig. 5d, e). However, no differences were found in the total number of OT-I T cells responding to antigen (Fig. 5e). The Nur77-GFP signal reports TCR triggering but not inflammation, as no GFP^+^ cells were detected upon antigenic challenge with VV that did not express OVA (Supplementary Fig. 5B). OT-I activation first occurred in the lungs, subsequently in the mediastinal LN (medLN) after 24 h, but was not observed in the spleen in the first 2 days (Supplementary Fig. 5C). In the lungs, all Nur77^+^ OT-I T cells were located in the IV^−^ fraction, indicating that antigen encounter took place within the lung tissue and not in the vasculature (Fig. 5f).

T_CM_ cells (CD127^+^ KLRG1^−^ CD62L^+^) were reported to traffic into inflamed tissue due to extensive synthesis of core 2 O-glycans that generate functional ligands for E- and P-selectins.^43^ Furthermore, T_EM_ cells (CD127^+^ KLRG1^−^ CD62L^−^) express more core 2 O-glycans than the more terminally differentiated effector-like CD127^−^ KLRG1^+^ CD62L^−^ T cells. As the MCMV-induced T cell pool consists of approximately 80% of KLRG1^+^ cells (Fig. 1c), we next determined which cells have the ability to migrate out of the vasculature. In the IV^−^ fraction of the MCMV-induced OT-I T cell pool, we detected comparable percentages of CD127^+^ KLRG1^−^, CD127^−^ KLRG1^+^ and CD127^−^ KLRG1^−^ cells, whereas in the influenza virus setting, hardly any CD127^−^ KLRG1^+^ cells were detected (Fig. 5g, h). These results confirm that CD127^+^ KLRG1^−^ cells are superior in trafficking into inflamed tissues compared to CD127^−^ KLRG1^+^, as the CD127^−^ KLRG1^+^ subset is the dominant subset in the vasculature (Supplementary Fig. 5D). CD127^−^ KLRG1^−^ OT-I cells, perhaps early stage effector cells, were also abundantly found in the IV^−^ fraction in both the MCMV and influenza virus setting. Thus, in MCMV-primed mice, not only the most abundant CD127^−^ KLRG1^+^ OT-I T cell population migrated out of the vasculature into the lung tissue, but also some smaller populations. tSNE analysis revealed that antigen recognition occurred in the IV^−^ fraction and that in the MCMV setting both CD127^+^ KLRG1^−^ and CD127^−^ KLRG1^+^ OT-I T cells responded to antigen (Fig. 5i). The Nur77 signal completely overlapped with CD69 expression. This is not surprising, as CD69 is an early activation marker for T cells upon TCR triggering. Some Nur77 signal overlapped with CD69^+^ CD103^+^ cells, indicating that also T_RM_ cells were responding to antigen. Interestingly, there was not such a clear separation for the MCMV- and influenza virus-induced T cells as observed in Fig. 1h, implying that the OT-I cells found in the lungs resemble each other more (Fig. 5i).

To test whether both CD127^+^ KLRG1^−^ and CD127^−^ KLRG1^+^ OT-I cells contributed to protection from a VV-OVA challenge, we sorted these subsets from MCMV-*ie2*-SIINFEKL-infected mice. Equal cell numbers were separately transferred into infection-matched recipients that were infected with MCMV-*ie2*-GP33 not expressing the OVA_257–264_ epitope, to achieve a similar pro-inflammatory environment without an endogenous OVA_257–264_/K^b^-specific T cell response (Fig. 5j). Mice that received either CD127^+^ KLRG1^−^ or CD127^−^ KLRG1^+^ OT-I cells similarly controlled a local VV-OVA challenge (Fig. 5k). Furthermore, no differences were found in the total number OT-I T cells recovered from the lungs (Fig. 5l). These data indicate that when present in equal numbers, both CD127^+^ KLRG1^−^ and CD127^−^ KLRG1^+^ provide protection from a local VV challenge.

### Hematopoietic cells in the lungs are infected by VV-OVA

**Fig. 6:**
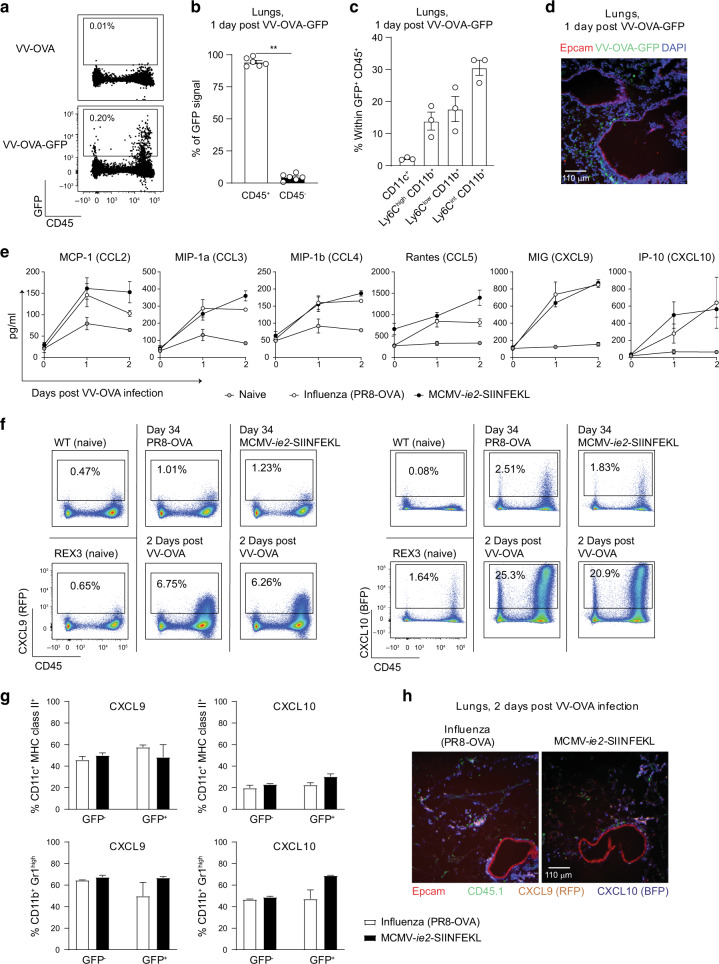
Hematopoietic cells in the lungs are infected by VV-OVA. Mice were infected with 5 × 10^6^ PFU VV-OVA or VV-OVA-GFP. 24 h post-infection, the lungs were isolated. **a** Flow cytometry plots show GFP expression versus CD45. **b** Bar graph shows percentage of GFP signal in the hematopoietic (CD45^+^) and non-hematopoietic (CD45^−^) compartment as mean + SEM (*n* = 6, pooled from two independent experiments). Two-sided Mann–Whitney test was used to determine statistical significance: **P < 0.01. **c** The distribution of the GFP-signal amongst the CD45^+^ compartment is shown as mean + SEM (*n* = 3, one out of two independent experiments is shown). **d** Microscopy image shows the lungs 1 day post infection with VV-OVA-GFP. **e** Concentration of different chemokines in lung homogenates is shown as mean ± SEM upon i.t. challenge with VV-OVA (*n* = 3, one out of two experiments is shown) in naive, influenza virus- and MCMV-infected mice. Day 0 represent day 28 post primary infection. **f** Flow cytometry plots show CXCL9 (RFP) and CXCL10 (BFP) production versus CD45 in REX3 mice. A WT and a naive REX3 mouse are included to determine the background level. **g** Bar graphs show the percentage of CXCL9 and CXCL10 that is produced by CD11c^+^ MHC class II^+^ and CD11b^+^ Gr1^high^ cells in non-infected (GFP^−^) and VV-infected (GFP^+^) cells in the lungs 2 days post-infection with 5 × 10^6^ PFU VV-OVA-GFP. **h** Microscopy image shows the lungs of REX3 mice 2 days post infection with 5 × 10^6^ PFU VV-OVA.

We next addressed which cells in the lungs are infected by VV-OVA. For this purpose, we generated a VV-OVA virus that also expresses GFP. Upon i.t. administration of this virus, the majority of the GFP signal was detected in CD45^+^ hematopoietic cells (Fig. 6a, b), mostly in CD11b^+^ cells (Fig. 6c). VV-OVA-GFP-infected cells were also found in the lung parenchyma, corresponding to the location where OT-I T cells were observed upon VV-OVA infection (Figs. 5a and 6d).

Leukocytes can be attracted to inflammatory sites by chemokines which, together with integrin-mediated binding, facilitate the migration through the endothelium to the site of infection.^44^ A number of chemokines including CCL2, CCL3, CCL4, CCL5, CXCL9, and CXCL10, were detected in lung homogenates upon VV-OVA infection. In mice that experienced an infection before the VV-OVA challenge, the chemokine concentrations were consistently higher as compared to VV-OVA infected naive mice (Fig. 6e). Correspondingly, the number of CD8 T cells (excluding the CD45.1^+^ OT-I T cells) in the IV^−^ fraction was larger in memory compared to naive mice (Supplementary Fig. 6). No marked differences were found in the chemokine induction between MCMV- or influenza virus-experienced mice. We confirmed the production of CXCL9 and CXCL10 upon VV-OVA infection using REX3 reporter mice that express red fluorescent protein (RFP) and blue fluorescent protein (BFP) under the control of the CXCL9 and CXCL10 promoter and found that hematopoietic cells, specifically CD11c^+^ MHC class II^+^ and CD11b^+^ Gr1^high^ cells, were the main producers of both chemokines in both VV-infected and non-infected cells (Fig. 6f, g). CXCL9-producing and CXCL10-producing cells were localized in the lung parenchyma and lining the Epcam^+^ airways (Fig. 6h). CD45.1^+^ OT-I T cells co-localized to these CXCL9^+^ and CXCL10^+^ producing cells (Fig. 6h). Combined, these results show that upon a respiratory VV-OVA infection, hematopoietic cells are the main infected cells and that both infected and non-infected hematopoietic cells produce chemokines.

### Chemokines are in part responsible for the extravasation of memory T cells into the lung tissue

**Fig. 7:**
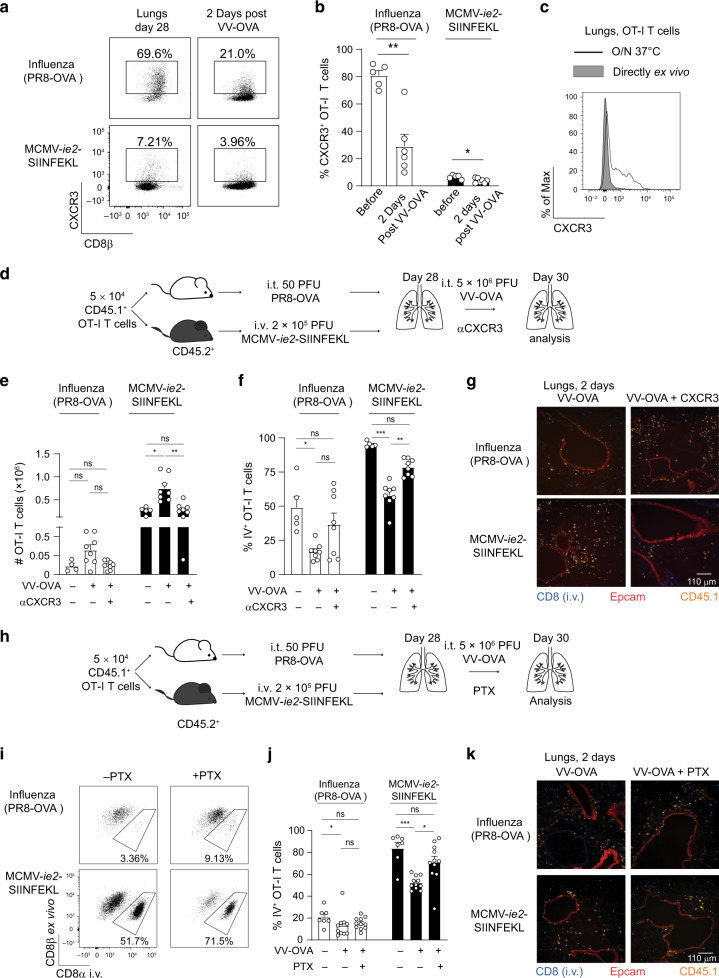
Chemokines are partially involved in the extravasation of memory T cells into the lungs. **a** Flow cytometry plots show expression of CXCR3 on OT-I T cells isolated from the lungs, 28 days post primary infection (left) or 2 days post rechallenge with VV-OVA (right). **b** Bar graphs show percentage of OT-I T cells expressing CXCR3 in the lungs, as mean + SEM (*n* = 5–6, pooled from two independent experiments). Each dot represents an individual mouse. **c** Histogram shows CXCR3 expression of OT-I T cells in the lungs either directly ex vivo or after overnight incubation at 37 °C. Cells are isolated from an MCMV experienced mouse 2 days post infection with 5 × 10^6^ PFU VV-OVA. **d** Experimental setup: 5 × 10^4^ CD45.1^+^ OT-I T cells were adoptively transferred into CD45.2^+^ hosts that were either infected i.t. with 50 PFU PR8-OVA or i.v. with 2 × 10^5^ PFU MCMV-*ie2*-SIINFEKL. Twenty-eight days post-infection, mice were i.t. challenged with 5 × 10^6^ PFU VV-OVA. Mice received 250 μg αCXCR3 blocking antibodies one day prior and during the rechallenge period. **e** Bar graphs show the total number of OT-I T cells recovered from the lungs as mean + SEM (*n* = 4–8, pooled from two independent experiments). **f** Bar graphs show the percentage of OT-I T cells associated with the vasculature determined by i.v. injection of 5 μg fluorescently conjugated αCD8 antibody 3 min prior to euthanasia (*n* = 4–8), pooled from two independent experiments). **g** Microscopy images show the lung 2 days post infection with 5 × 10^6^ PFU VV-OVA. **h** Experimental setup: 5 × 10^4^ CD45.1^+^ OT-I T cells were adoptively transferred into CD45.2^+^ hosts that were either infected i.t. with 50 PFU PR8-OVA or i.v. with 2 × 10^5^ PFU MCMV-*ie2*-SIINFEKL. Twenty-eight days post-infection, mice were i.t. challenged with 5 × 10^6^ PFU VV-OVA. Mice received 400 ng PTX i.p. on the day of and one day after VV-OVA rechallenge. **i** Flow cytometry plot shows in vivo CD8α labeling and ex vivo CD8β staining on OT-I T cells in the lungs 2 days post VV-OVA infection upon injection with 5 μg fluorescently conjugated αCD8 antibody 3 min prior to euthanasia. **j** Bar graphs shows the percentage of IV^+^ OT-I T cells in the lungs 2 days post VV-OVA infection (*n* = 7–11, pooled from three independent experiments) as mean + SEM. **k** Microscopy images show the lung 2 days post infection with 5 × 10^6^ PFU VV-OVA. Two-sided Mann–Whitney test **b** or Kruskal–Wallis test with Dunn’s post hoc (**e**, **f**, **j**) test was used to determine statistical significance: *P < 0.05, **P < 0.01, ***P < 0.001 and not significant (ns) *P* ≥ 0.05.

CXCL9 and CXCL10 bind to the chemokine receptor CXCR3. Although there was no difference in the induction of CXCL9 and CXCL10 in MCMV- and influenza virus-experienced mice, CXCR3 was differentially expressed on OT-I T cells. The majority of lung-localized influenza virus-induced memory OT-I T cells expressed high levels of CXCR3, whereas CXCR3 was hardly detected on MCMV-induced OT-I T cells (Fig. 7a, b). CXCR3 expression was, however, detected on a subset of MCMV-induced OT-I T cells in the spleen (Supplementary Fig. 7A) and correlated with the expression of CD127 (Supplementary Fig. 7B). Upon VV challenge, OT-I T cells quickly downregulated CXCR3 (Fig. 7a, b). Although hardly any CXCR3-expressing cells were found directly ex vivo in the MCMV-setting, overnight incubation at 37 °C of lung single cell suspensions revealed a proportion of cells expressing CXCR3, indicating that CXCR3 is actively downregulated within the lungs (Fig. 7c and Supplementary Fig. 7C). CXCR3 upregulation was hardly observed on OT-I T cells obtained from the spleen after overnight incubation (Supplementary Fig. 7D). Next, we determined the importance of CXCR3 signaling on T cell recruitment and positioning in the lungs (Fig. 7d). Blockade of CXCR3 during the VV-OVA challenge diminished the number of OT-I T cells that was found in the lung after VV-OVA challenge in MCMV-experienced mice (Fig. 7e), and the fraction of OT-I T cells associated with the vasculature was increased (Fig. 7f). A similar trend was observed in the influenza virus setting (Fig. 7e, f). In both settings, OT-I T cells were still found in the lung parenchyma and the positioning of these cells was not altered (Fig. 7g). Furthermore, no differences in the viral load were observed upon CXCR3 blockade (Supplementary Fig. 7E). These data show that CXCR3 signaling is in part involved in recruitment of memory OT-I T cells to the lungs and from the vasculature into the lung parenchyma. However, in the absence of CXCR3 signaling, there are still sufficient numbers of antigen-specific cells present in, or recruited into the lung parenchyma to control VV-OVA replication.

Signaling via chemokines occurs via G-protein-coupled receptors, and this signaling is inhibited by pertussis toxin (PTX). As we observed a large range of different chemokines produced upon VV-OVA challenge, mice were administrated with PTX during the VV-OVA challenge period to inhibit all chemokine receptor signaling (Fig. 7h). PTX treatment partially inhibited the migration of MCMV-induced OT-I T cells from the vasculature into the lung tissue (Fig. 7i, j). However, no difference in the viral load was observed upon PTX treatment (Supplementary Fig. 7F). Similarly to CXCR3 blockade, the repositioning of the OT-I T cells to the lung parenchyma remained normal in both the MCMV- and the influenza virus-setting (Fig. 7k). These data indicate that chemokines are, at least in part, responsible for the recruitment and relocation of memory OT-I T cells from the vasculature deeper into the lung tissue, but other mechanisms also operate to control VV-OVA replication.

## Discussion

Memory CD8 T cells residing in peripheral tissues are of interest in the context of vaccination. The memory T cell subset providing the best protection in a given infection may vary. By using distinct viral infections, we induced memory T cell pools with unique characteristics within the lung tissue and compared their protective capacity towards a local VV challenge. Upon influenza virus infection, the majority of the established memory T cells had a T_RM_ phenotype, whereas lung localized MCMV-specific CD8 T cells predominantly expressed KLRG1 and thus exhibited an effector-like phenotype. Factors that contribute to the induction of a specific phenotype include the route and dose of infection, the viral tropism and the pro-inflammatory environment. For instance, only respiratory and not systemic administration of MCMV or influenza virus results in the formation of lung T_RM_ cells.^34,35,45^ Moreover, antigen is required for the maintenance of the MCMV-specific inflationary T cell pool and the MCMV inoculum dose impacts on the balance between CD127^+^ and KLRG1^+^ MCMV-specific CD8 T cells found within the lung tissue.^27,46,47^ Differences in the localization between MCMV- and influenza virus-induced memory T cells were also observed. CD103 tethers T_RM_ cells to E-cadherin expressed by epithelial cells, likely explaining their localization in close proximity to epithelial structures. MCMV-specific KLRG1^+^ cells express CX3CR1,^48^ which could explain their localization to the vasculature.

Despite their differences in anatomical localization, MCMV- and influenza virus-induced memory T cells both provided protection from a local respiratory VV infection. We detected VV mostly in hematopoietic cells. It is plausible that upon infection with other pathogens, a different outcome in protective capacity may be observed. For instance, intra-nasal administration of MCMV vectors encoding influenza virus epitopes facilitates the clearance of influenza virus in a T_RM_-dependent manner.^35^ Despite having large number of effector-like cells in the lungs, mice without T_RM_ cells had a higher viral load in the lungs, indicating that in an influenza virus setting, T_RM_ cells are superior in protection as compared to circulating memory T cells. Furthermore, T_RM_ cells induced by intra-nasal administration of MCMV-vectors encoding respiratory syncytial virus (RSV) epitopes also enhanced the protection towards RSV infection, compared to a setting in which only circulatory memory T cells were present.^34^ As both influenza virus and RSV infect alveolar epithelial cells^49–51^ and T_RM_ cells are lining the epithelial walls, their anatomical localization could give them an advantage over vasculature-associated memory CD8 T cells induced by systemic administration of MCMV.

To control VV replication, effector cytokines such as IFN-γ and TNF are critical.^38,39^ Although we observed a decrease in the number of lung resident memory CD8 T cells 60 days post-influenza virus infection, the remaining population was still protective. It could be that very few memory T cells are actually needed to provide protection in our experimental setup. In Fig. 5k, only 500 cells were recovered from the lungs after VV-OVA challenge, and a slight, but significant protective effect was observed compared to mice without any memory OT-I T cells. It has been shown before that the number of VV-specific memory CD8 T cells in the lung corresponds to their protective capacity.^52^ Of note, the number of cells responding to secondary VV-OVA antigen exposure, quantified by Nur77-GFP expression, did not differ between influenza virus- and MCMV-immune mice (Fig. 5e). IFN-γ can be sensed up to a distance of 800 μm from its producer in tumor tissue,^53^ indicating that IFN-γ can act beyond the cells that are in direct contact with T cells. Whether this range is achieved in case of respiratory VV infections remains to be determined. In any case, we readily detected the IFN-γ-induced chemokines CXCL9 and CXCL10 throughout the lung tissue in REX3 mice.

We found that upon local inflammation, MCMV-induced T cells extravasate in an antigen-independent manner and chemokines played a partial role in this process. Integrin-mediated extravasation likely contributes as well.^44^ In the MCMV setting, the increase in the lung localized OT-I T cell pool was mostly due to T cell recruitment, whereas in the influenza setting both T cell recruitment and proliferation likely contributed. In the female reproductive tract, T_RM_ cell activation recruits other cells of the immune system into the tissue, including bystander memory T cells, via IFN-γ-mediated upregulation of VCAM-1 and by local induction of CXCL9 and CXCL10.^14,54^ Also in the lungs, T_RM_ cell activation facilitates the accumulation of memory CD8 T cells upon influenza virus infection.^35^ In our experimental setup, migration of antigen-specific memory T cells from the vasculature into the lung parenchyma was more pronounced in the MCMV as compared to the influenza virus setting, likely due to the large numbers of memory cells that are present in the circulation upon MCMV infection. We observed a similar recruitment of bystander CD8 T cells into lung parenchyma in both the MCMV and influenza virus setting (Supplementary Fig. 6). Lung-localized MCMV-induced T cells were potent IFN-γ producers, and both CXCL9 and CXCL10 were produced upon VV-OVA infection. Recently, it was shown that T_RM_ cells in the female reproductive tract and the skin maintain their proliferative capacity.^40,41^ Here we observed local proliferation of memory T cells in the lungs upon antigen exposure in the influenza virus setting. However, whether the T_RM_ cells or other memory T cell subsets were proliferating remains to be determined.

Upon lung inflammation, both MCMV-specific KLRG1^+^ and CD127^+^ OT-I T cells extravasated from the vasculature. CD127^+^ cells were superior in entering the lung tissue as a higher percentage of CD127^+^ cells was found within the lung tissue as compared to the blood. This is likely due to higher Core 2 O-glycan synthesis in CD127^+^ cells that enables these cells to traffic into inflamed tissue.^43^ Both CD127^+^ and KLRG1^+^ cells were able to control VV-infection in the lungs. We have shown before that the number of KLRG1^+^ antigen-specific cells in the ovaries correlated with the degree of protection towards an i.p. VV challenge.^2^ These differences might be explained by tissue-specific factors, as the lung is highly vascularized. Furthermore, the ovaries are the preferred site of VV replication and therefore VV might infect also non-hematopoietic cells in this organ.

In many infection and vaccination settings, CXCR3 expression is required for T cells to enter the lung tissue.^55,56^ Lung entry subsequently affects CXCR3 expression in an antigen-dependent manner^57^ and CXCR3^low^ cells are preferentially found in close proximity to the vasculature.^57^ However, both CXCR3^high^ and CXCR3^low^ cells can provide protection from respiratory infections.^56–59^ The signals required for the downregulation of CXCR3 are not entirely clear. We found that MCMV-induced memory T cells in the lung do not express CXCR3 ex vivo, but upon overnight incubation its expression was restored, likely indicating that the lung environment actively promotes CXCR3 downregulation. Blocking of CXCR3 during the rechallenge period diminished the number of cells recovered from the lung, indicating that CXCR3 is at least in part involved in the recruitment of memory OT-I cells to the lung. In the MCMV setting, CXCR3 is mostly expressed by CD127^+^ cells (Supplementary Fig. 7B), potentially recruiting these cells upon VV-OVA challenge.

MCMV-induced memory T cells are stably maintained over time, making CMV-vectors an interesting candidate for T cell-based vaccines. Until day 60 post-infection, we did not observe a decrease in the protective capacity of influenza virus-induced memory T cells, despite a reduction in T_RM_ cells. It is conceivable that a loss in protective capacity would become evident at later time points after influenza virus infection. Here we set out to determine which cells are superior in providing early protection from a local viral challenge. This likely depends on the timing of secondary infection as well as on the local tropism of the recall pathogen. For future vaccine approaches aimed at eliciting T cells at mucosal sites, it is important to consider which memory T cell subset provides protection from a specific pathogen and therefore should be induced by the vaccines. Our data show that in case of a respiratory VV infection, both T_RM_ cells and MCMV-induced circulatory cells fulfill this requirement.

## Methods

### Ethics statement

This study was conducted in accordance to the guidelines of the animal experimentation law (SR 455.163; TVV) of the Swiss Federal Government. The protocol was approved by Cantonal Veterinary Office of the canton Zürich, Switzerland (Permit number 168/2015, 114/2017 and 58/2020).

### Mice

C57BL/6J were purchased from Janvier Elevage and were used as WT mice. The congenic CD45.1 (Ly5.1) and the C57BL/6-Tg(TcraTcrb)1100Mjb/J (OT-I) mice were bred in house. Nur77-GFP OT-I mice were generated by crossing the OT-I mouse with the Nur77-GFP reporter mouse.^42^ REX3 mice expressing RFP and BFP under the control of the CXCL9 and CXCL10 promoter were obtained from Prof. Dr. Matteo Iannacone and are described.^60^ All mice were housed and bred under specific pathogen-free facilities at the Eidgenössische Technische Hochschule (ETH) Hönggerberg. All mice were between 7 and 12 weeks at the start of each experiment and were age- and sex-matched.

### Viruses

The recombinant influenza A virus (IAV) strain A/PR8 expressing OVA_257-264_^61^ was a kind gift from Stephen Turner (St. Jude Children’s Research Hospital, Memphis, USA) and was grown for 2 days at 35 °C in the allantoic cavities of 10–11-day-old fertile chicken eggs. Viral titers were quantified by standard plaque assay using Madin–Darby canine kidney cells. Mice were infected with 50 PFU intra tracheally (i.t.) in 50 µl PBS. For an i.t. infection mice were anesthesized by isoflurane inhalation or by an i.p. injection with 5 µg xylazine/100 µg ketamine per gram of body weight.

MCMV-*ie2*-SIINFEKL and MCMV-*ie2*-GP33 are obtained from Dr. L. Cicin-Sain, contain the m157 gene and are described.^2,62,63^ Mice were infected with 2 × 10^5^ PFU either i.v. or i.t.. MCMV stocks were propagated on M2-10B4 cells. The virus was subsequently purified by ultracentrifugation using a 15% sucrose cushion. Viral titers were determined by standard plaque assay as described.^64^ Recombinant VV (Western Reserve) expressing Ovalbumin protein (VV-OVA) inserted into the thymidine kinase gene was grown on BSC40 cells and was provided by Dr. P. Klenerman (Oxford Univeristy). Recombinant VV expressing the LCMV glycoprotein (VV-G2) was originally obtained from Dr. D.H.L. Bishop (Oxford University) and was grown on BSC40 cells at low multiplicity of infection. Recombinant Western Reserve vaccinia Ovalbumin nuclear-eGFP virus (WR OVA NP-eGFP, VV-OVA-GFP) was generated by inserting pNP-SIINFEKL-eGFP into the A56L locus of Western Reserve vaccinia Ovalbumin virus (WR VV-OVA) using homologous recombination as described previously.^65–67^ Briefly, BSC40 cells were infected with VV-OVA WR, transfected with linearized plasmid 4 h post infection and harvested 48 h post infection. Clonal recombinant fluorescent virus was selected though multiple serial dilution infection rounds. Mice were challenged i.t. with 5 × 10^6^ PFU VV (unless stated otherwise) in 50 µl under anasthesia as described.

### Plaque assays

To determine the viral load in the lungs of VV-infected mice, 1.5 × 10^5^ BSC40 cells were plated per well of a 24-well plate in MEM supplemented with 5% FCS, penicilin, streptamycin, and glutamin. The following day, lungs of VV-infected mice were lysed two times for 1.5 min at 25 Hz using a tissue lyser (Qiagen). Samples were centrifuged for 5 min at 3.5×*g* and supernatant was used. 200 µl of serial dilutions of homogenized lung lysates were propagated on BSC40, upon which the plates were incubated for 1 h at 37 °C. 1 ml of complete MEM medium was added and plates were incubated for another day at 37 °C. Plates were fixed for 20 min by adding 200 µl of 4% formalin and subsequently developed using crystal violet solution.

### Flow cytometry

Single cell suspensions were prepared from spleen by meshing the tissue through a 70 µM cell strainer. Erythrocytes were lysed using a hypotonic ammonium-chloride–potassium buffer for 1 min. To prepare a single cell suspension from the lungs, mice were perfused with PBS to remove all blood-associated cells, the tissue was cut into small pieces, subsequently incubated with Collegenase I and DNAse I for 45 min, followed by a 30% percoll gradient. To determine the lung tropism of VV-OVA-GFP or the production of CXCL9 and CXCL10, no percoll gradient was used. Cells were incubated with fluorescently conjugated antibodies for 30 min at 4 °C. Dead cells were excluded using a LIVE/DEAD fixable NEAR-IR staining. For intracellular Ki67 staining, the FoxP3 kit (invitrogen) was used according to manufacterer’s protocol. Multi-parametric flow cytometric analysis was performed using LSRII flow cytometer (BD Biosciences) or LSRFortessa (BD Biosciences) with FACSDiva software. Data was analyzed using FlowJo software (Tree Star). Cells were sorted using a FACS Aria sorter.

### Chemokine bead assay

To determine the concentration of different chemokines in the lungs, mice were perfused with PBS, lungs were snap-frozen in liquid nitrogen and stored at −80 °C until further use. Lungs were homogenized in 1 ml of MEM supplemented with 5% FCS, penicilin, streptamycin, and glutamin and were lysed two times for 1.5 min at 25 Hz using a tissue lyser (Qiagen). Samples were centrifuged for 5 min at 3.5×*g* and supernatant was used. To determine the chemokine concentration the mouse pro-inflammatory chemokine panel (Biolegend) was used with v-bottom plate according to manufacturers protocol. Data was analzed with the Legendplex analysis software (Biolegend).

### MHC class I tetramers and in vitro restimulations

MHC class I monomers for OVA_257-264_ (K^b^ restricted) were produced as described^68^ and were tetramerized using streptavidin–APC. For in vitro restimulation, cells were stimulated for 5 h at 37 °C with 1 µg/ml OVA_257-264_ peptide in the presence of 2 µg/ml Brefeldin A. Cell surface staining was performed as described, and cells were subsequently fixed with 0.5% PFA overnight. The following day, intracellular cytokine staining was performed by diluting the antibodies in perm/wash buffer (Life Technologies) and incubating the samples for 30 min at 4 °C.

### Adoptive transfer, FTY720, PTX, CXCR3 blockade and BrdU treatment

OT-I or Nur77 OT-I CD8 T cells were enriched from spleens by negative selection according to manufacterer’s protocol (Mojosort, biolegend). 5 × 10^4^ TCR transgenic OT-I cells were adoptively transferred into new hosts that were subsequently infected with influenza virus or MCMV-expressing OVA_257-264_. For microscopy analysis, 1 × 10^5^ OT-I T cells were transferred into naive recipients prior to infection. To inhibit T cell recruitment from secondary lymphoid organs, mice received 25 µg FTY720 on day 0 and 1 of VV-OVA infection i.p. and starting at day 0, FTY720 was administrated in the drinking water at a concentration of 5 µg/ml. To inhibit chemokine receptor signaling, mice received 400 ng PTX i.p. on the day and one day after VV-OVA challenge. For CXCR3 blockade, mice received 250 μg αCXCR3 antibodies (clone CXCR3-173, BioXCell) i.p. on day −1, 0, and 1 post VV-OVA challenge. To determine proliferation, mice received 0.8 mg/ml BrdU in the drinking water starting on the day of rechallenge. BrdU staining was performed according to manufacturer’s protocol for BrdU flow kit (BD).

### Immunofluorescence microscopy

To fix the lungs, 1 ml of 1% PFA was infused into the lungs of euthanized mice with a cannula inserted via a small incision in the trachea. After 20 min, PFA was removed and was replaced by 1 ml of 20% sucrose. After an additional incubation of 20 min, sucrose was replaced by 1 ml of optimum cutting temperature (OCT). The lungs were removed, frozen in liquid nitrogen and stored at −20 °C until further use. Cryosections of 10 µM were made and air dried. For the staining, slides were re-hydrated in PBS and blocked with 10% rat serum in PBS. Slides were stained with antibodies diluted in PBS containing 1% rat serum and mounted with Mowiol. Within 1 day, images were acquired on a Visitron confocal system inverse confocal microscope with ×10 magnification. Data was analyzed using Volocity software.

### Statistical analysis

Graphpad prism 8.0 software was used to calculate significance between samples. *P*-values < 0.05 were considered as significant. Statistical test is indicated in each figure.

## Supplementary information

Supplementary figures
