# Supplementary material for: Influenza- and MCMV-induced memory CD8 T cells control respiratory vaccinia virus infection despite residence in distinct anatomical niches
Source: Mucosal Immunol. 2021 Jan 21;14(3):728–42. doi: 10.1038/s41385-020-00373-4 (PMC8075924; doi:10.1038/s41385-020-00373-4)
Supplement: Supplementary file 1 — Supplementary figures [file 41385_2020_373_MOESM1_ESM.pdf]

Supplementary Figure 1

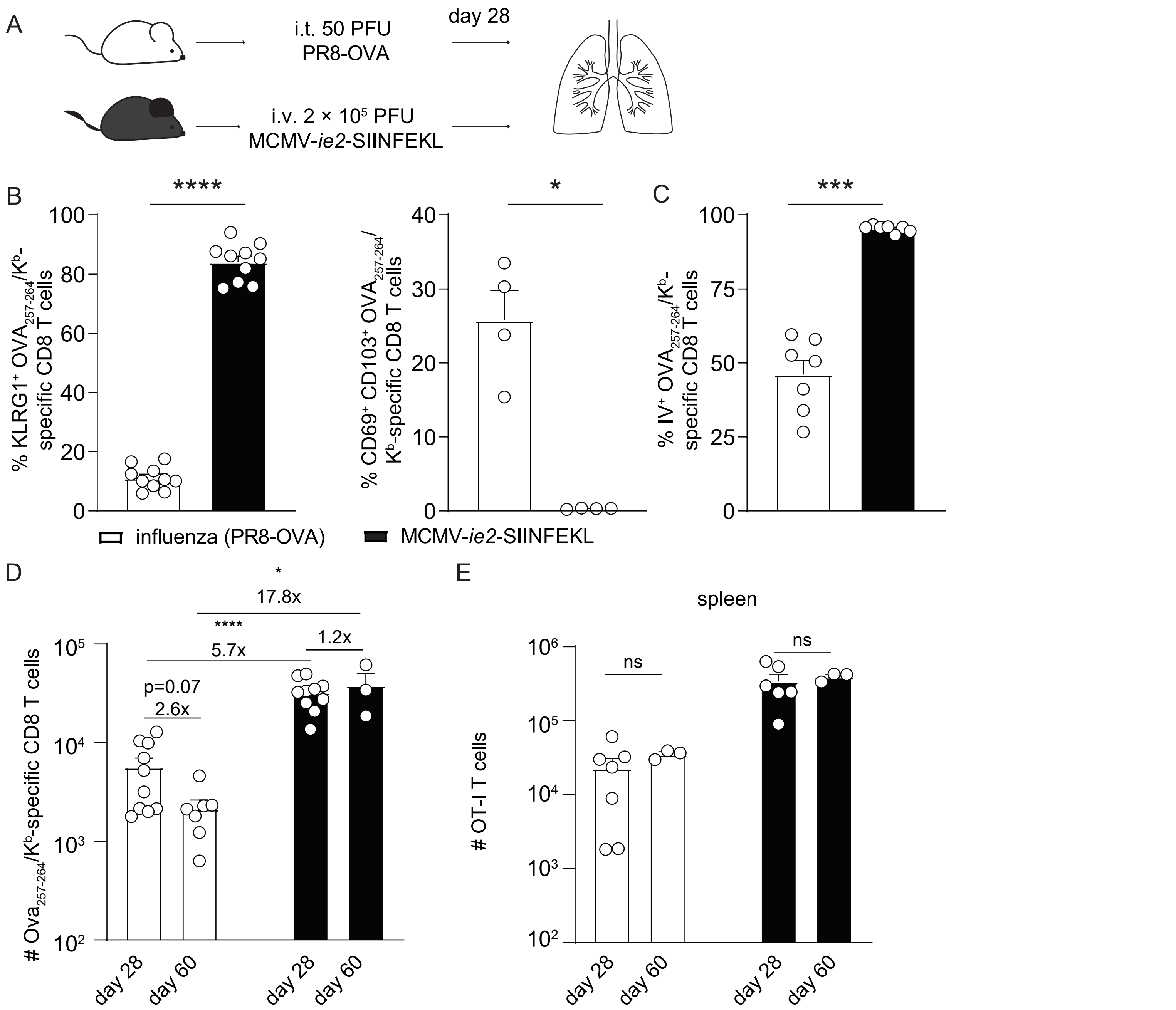

Supplementary Figure 1: MCMV and influenza virus infection induce distinct memory T cell pools in the lungs. (A) Experimental setup: mice were infected i.t. with 50 PFU PR8-OVA or i.v. with 2×10^5 PFU MCMV-*ie2*-SIINFEKL. Twenty-eight days post infection the phenotype of the endogenous $OVA_{257–264}/K^b$-specific CD8 cells in the lungs was determined using MHC class I tetramers. (B) Bar graphs show the percentage of $OVA_{257–264}/K^b$-specific CD8 T cells that expresses KLRG1 or CD69 CD103. For KLRG1 pooled data from three independent experiments is shown (n=10). For CD69 CD103 one representative out of three independent experiments is shown (n=4). (C) Mice received 5 µg fluorescently conjugated αCD8 antibody 3 minutes prior to euthanasia. Bar graphs indicate the percentage of cells stained by the i.v. injected CD8 antibody (n=7, pooled from two independent experiments). (D) The total number of $OVA_{257–264}/K^b$-specific CD8 T cells in the lungs at day 28 and day 60 post-infection. Fold difference is indicated. Pooled data from three independent experiments is shown for day 28, and 1 experiment for day 60 (n=3-10). (E) 5×10^4 $CD45.1^+$ OT-I T cells were adoptively transferred into $CD45.2^+$ hosts that were either infected i.t. with 50 PFU influenza-expressing-$OVA_{257-264}$ (PR8-OVA) or i.v. with 2×10^5 PFU MCMV-*ie2*-SIINFEKL. Twenty-eight and sixty days post infection, the number of OT-I T cells was determined in the spleen, pooled data from two independent experiments is shown for day 28, and 1 experiment for day 60 (n=3-7). All bar graphs show mean + SEM. Each dot represents an individual mouse. Statistical significance was determined using two-sided Mann-Whitney test with: *P<0.05, ***P<0.001, ****P<0.0001 and not significant (ns) $P \geq 0.05$.

Supplementary Figure 2

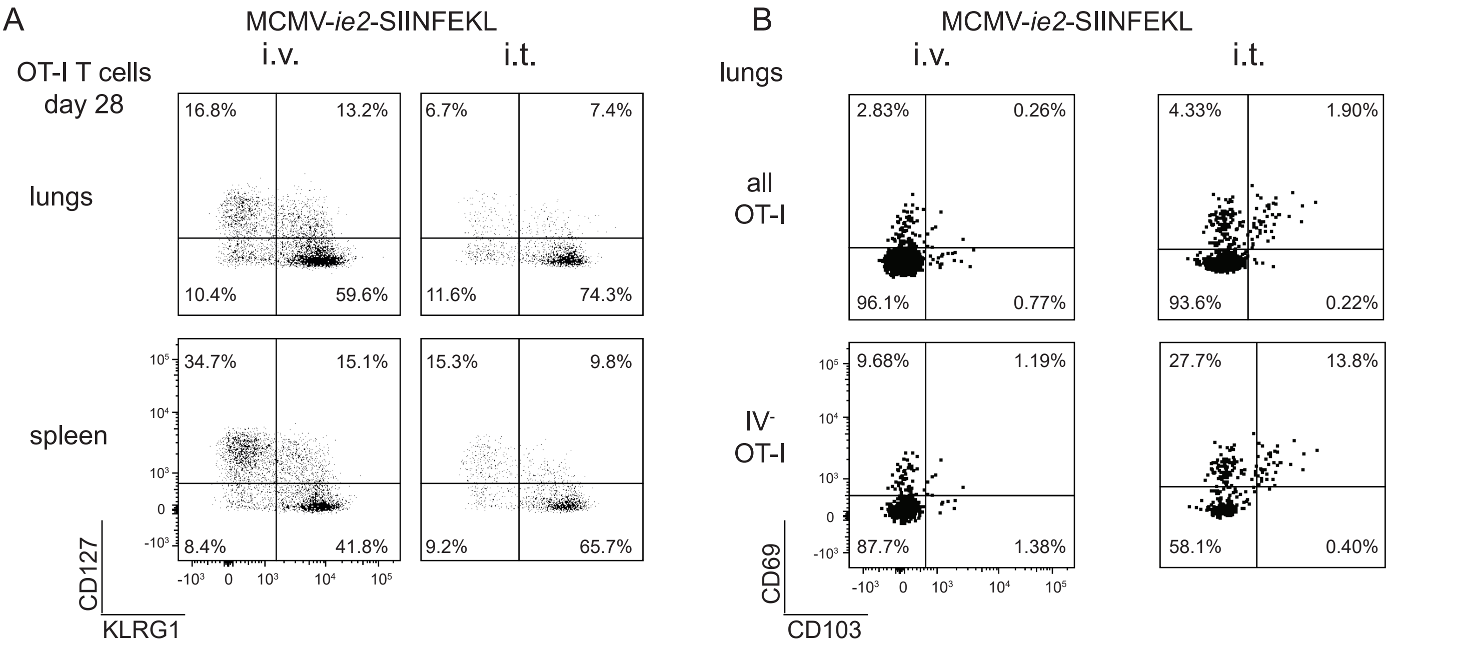

Supplementary Figure 2: Intra-tracheal infection with MCMV induces an effector memory T cell response in the lungs.

Mice received 5×10^4 OT-I T cells and were subsequently i.v. or i.t infected with 2×10^5 PFU MCMV-*ie2*-SIINFEKL. On day 28 post-infection the phenotype of the OT-I T cells was determined. (A) Representative plots show the expression of KLRG1 CD127 on OT-I T cells in the lungs and spleen. (B) Representative plots show the expression of CD69 CD103 on all, or on IV$^-$ OT-I T cells in the lungs.

Supplementary Figure 3

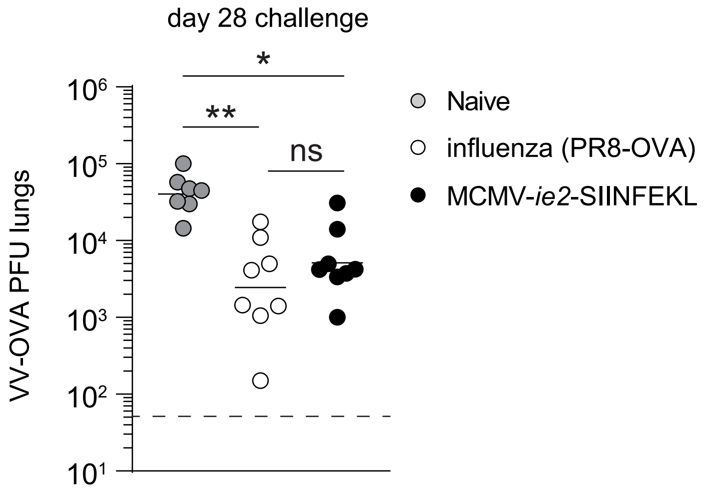

Supplementary Figure 3: MCMV- and influenza-induced memory T cells protect from a local vaccinia virus challenge in the lungs.

Mice were infected i.t. with 50 PFU PR8-OVA or i.v. with 2×10^5 PFU MCMV-*ie2*-SIINFEKL. On day 28 post primary infection mice were i.t. challenged with VV-OVA. Two days post challenge the viral load was determined in the lungs. A naive group of mice challenged with VV-OVA was also included. Pooled data from two independent experiments is shown (n=7-8). Each dot represents an individual mouse, geometric mean and limit of detection are indicated. Statistical significance was determined using Kruskal-Wallis test with Dunn's post-hoc test to correct for multiple comparisons: *$P<0.05$, **$P<0.01$ and not significant (ns) $P \geq 0.05$.

Supplementary Figure 4

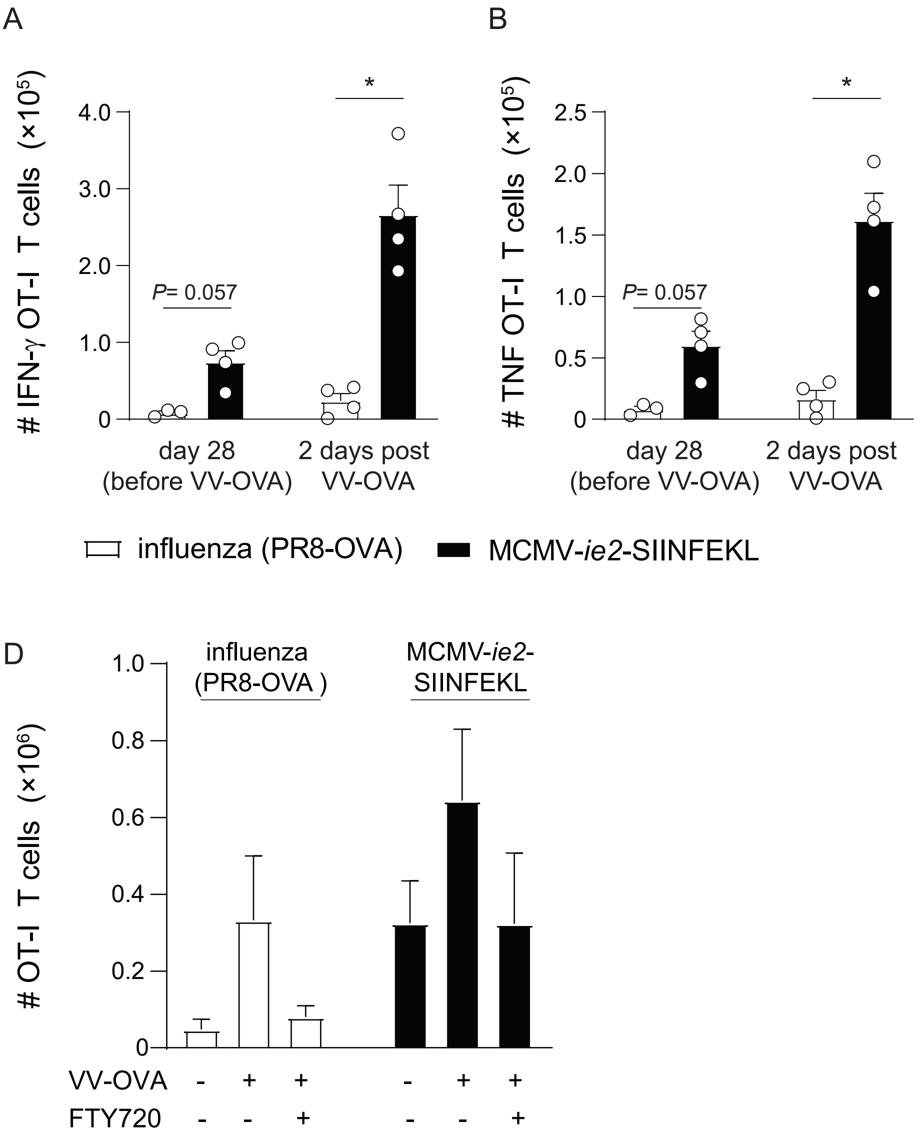

Supplementary Figure 4: MCMV infection induces a larger number of cytokine producing cells. Cytokine production was determined in OT-I T cells from the lungs of MCMV- or influenza virus-infected mice, before (28 days post primary infection) and two days post challenge with 5×10^6 PFU VV-OVA. Bar graphs show the number of OT-I T cells that produce IFN-γ (A), TNF (B) or IL-2 (C). (D) Mice were treated with FTY720 during the VV-OVA challenge period. Total number of OT-I T cells in the lungs is shown. All bar graphs represent mean + SEM and each dot represents an individual mouse (n=3-4). One experiment out of two independent experiments is shown. Mann-Whitney test (A-C) or Kruskal-Wallis test with Dunn's post-hoc test to correct for multiple comparisons (D) was used to determine significance: not significant $P \geq 0.05$, *$P<0.05$.

Supplementary Figure 5

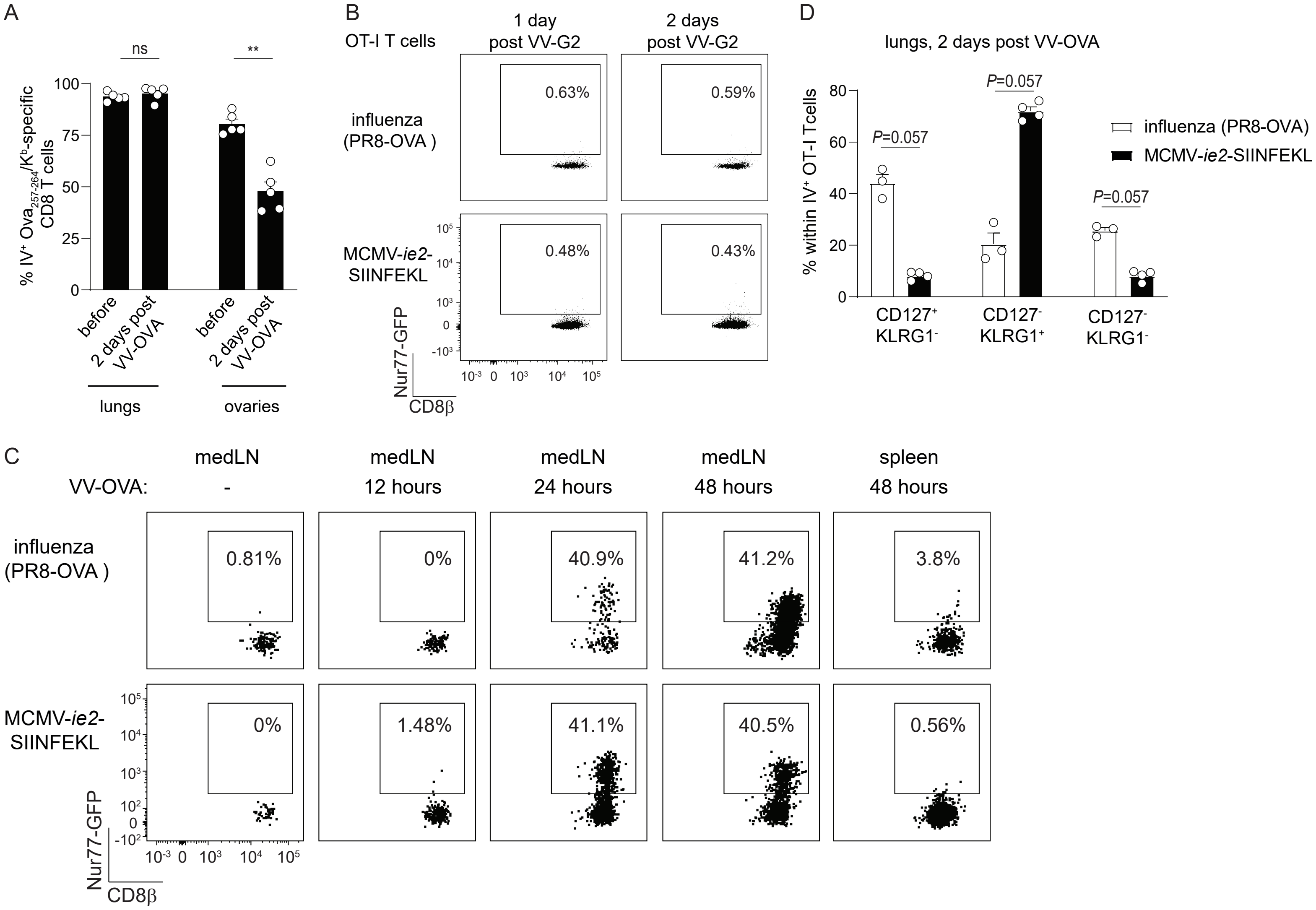

Supplementary Figure 5: The migration of MCMV-induced OT-I memory CD8 T cells out of the vasculature is dependent on local inflammation.

Mice were i.v. infected with 2×10^5 PFU MCMV-*ie2*-SIINFEKL. On day 30 post infection, 2×10^7 PFU VV-OVA was i.p. administrated. Three days post-secondary challenge, 5 µg of fluorescently conjugated αCD8 antibodies were administrated i.v. 3 minutes prior to euthanasia. The percentage of i.v. labelled $OVA_{257–264}/K^b$-specific CD8 T cells was determined in the lungs and the ovaries using MHC class I tetramers. Bar graphs represent mean + SEM and each dot represents an individual mouse (n=5). (B) 5×10^4 $CD45.1^+$ Nur77-GFP OT-I T cells were adoptively transferred into host mice that were subsequently infected i.t. with 50 PFU PR8-OVA or i.v. with 2×10^5 PFU MCMV-*ie2*-SIINFEKL. After 28 days, mice were i.t. challenged with 3×10^5 PFU VV-G2. Flow cytometry plots show Nur77 expression on OT-I T cells in the lungs 1 and 2 days post VV-G2 infection. (C) 5×10^4 $CD45.1^+$ Nur77-GFP OT-I T cells were adoptively transferred into host mice that were subsequently infected with 50 PFU i.t. PR8-OVA or i.v. with 2×10^5 PFU MCMV-*ie2*-SIINFEKL. After 28 days, mice were i.t. challenged with 5×10^6 PFU VV-OVA. Flow cytometry plots show Nur77 expression on OT-I T cells in the medLN and the spleen at several times post-infection. (D) 5×10^4 $CD45.1^+$ OT-I T cells were adoptively transferred into host mice that were subsequently infected i.t. with 50 PFU PR8-OVA or i.v. with 2×10^5 PFU MCMV-*ie2*-SIINFEKL. After 28 days, mice were i.t. challenged with 5×10^6 PFU VV-OVA. Two days post-secondary challenge, 5 µg of fluorescently conjugated αCD8 antibodies were administrated i.v. 3 minutes prior to euthanasia. The percentage of cells expressing CD127 and/or KLRG1 within the IV^+ fraction is shown (n=3-4, one out of three independent experiments is shown). Two-sided Mann-Whitney test was used to determine statistical significance: $^{**}P<0.01$, and not significant (ns) $P \geq 0.05$.

Supplementary Figure 6

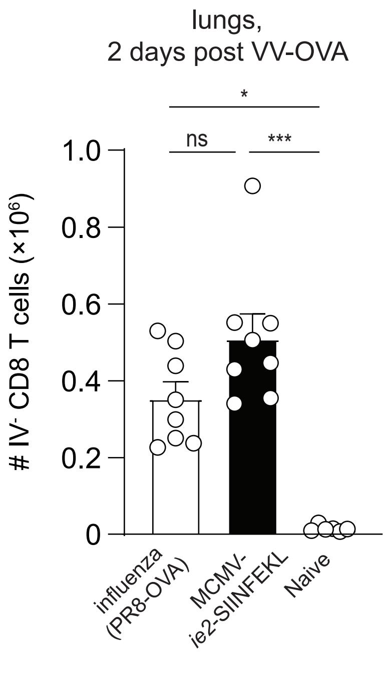

Supplementary Figure 6: Extravasation of CD8 T cells upon respiratory VV challenge.
5 × 10^4 $CD45.1^+$ OT-I T cells were adoptively transferred into $CD45.2^+$ hosts that were either infected i.t. with 50 PFU PR8-OVA or i.v. with 2 × 10^5 PFU MCMV-*ie2*-SIINFEKL. On day 28, mice received i.t. 5 × 10^6 PFU VV-OVA. Two days post challenge, the total number of CD8 T cells (excluding the OT-I T cells) in the IV^- fraction was determined by administration of a fluorescently conjugated αCD8 antibody three minutes prior to euthanasia. Pooled data from two experiments is shown (n=6-8). Statistical significance was determined using Kruskal-Wallis test with Dunn's post-hoc test to correct for multiple comparisons: *P<0.05, ***P<0.001 and not significant (ns) P ≥ 0.05.

Supplementary Figure 7

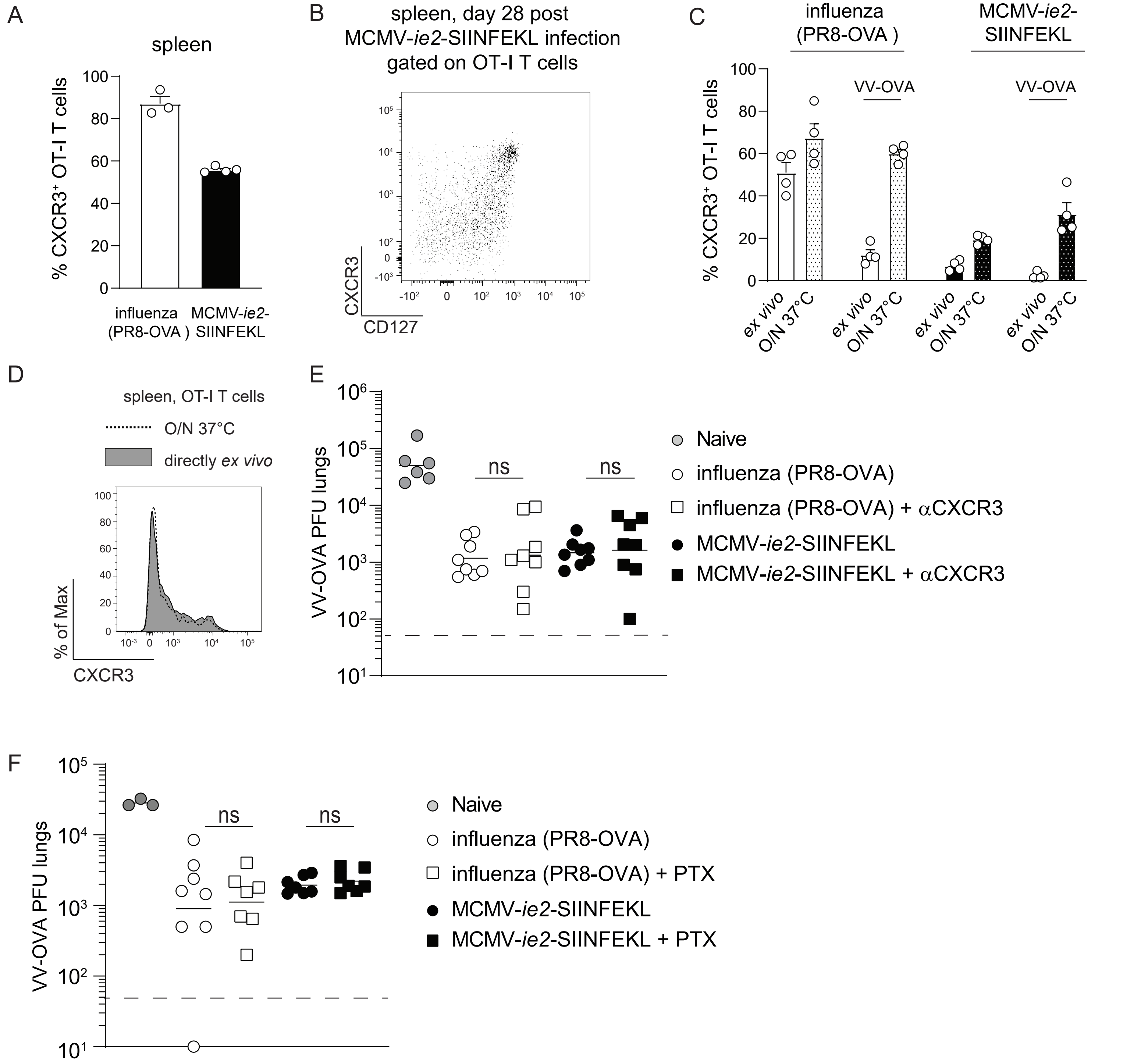

Supplementary Figure 7: Importance of chemokine signalling for the control of a respiratory VV-OVA challenge. (A) Bar graph shows CXCR3 expression on memory OT-I T cells in the spleen either induced by MCMV-*ie2*-SIINFEKL or influenza virus (PR8-OVA) (as described in Figure 1A) as mean + SEM (n=3-4, one out of three experiments is shown). (B) Flow cytometry plot shows CXCR3 and CD127 expression on OT-I T cells in the spleen, 28 days post infection with MCMV. (C) Bar graph shows CXCR3 expression on OT-I T cells in the lungs stained directly *ex vivo* or after overnight incubation at 37°C. Cells are isolated from influenza virus- or MCMV-experienced mice 28 days post-primary infection or two days post infection with 5×10^6 PFU VV-OVA, (n=4, one experiment out of 2 is shown). (D) Histogram shows CXCR3 expression of OT-I T cells in the spleen either directly *ex vivo* or after overnight incubation at 37°C. Cells are isolated from an MCMV experienced mouse two days post infection with 5×10^6 PFU VV-OVA. (E) Viral load of VV-OVA in the lungs 2 days post challenge in influenza virus- and MCMV-experienced mice with and without CXCR3 blockade is shown. Pooled data of two experiments is shown (n=6-8). Each dot represents and individual mouse, geometric mean and limit of detection are shown. (F) Viral load of VV-OVA in the lungs 2 days post challenge in influenza virus- and MCMV-experienced mice with and without PTX treatment is shown. Pooled data of two experiments is shown (n=3-8). Each dot represents and individual mouse, geometric mean and limit of detection are shown. Statistical significance was determined using Kruskal-Wallis test with Dunn's post-hoc test to correct for multiple comparisons: not significant (ns) $P \geq 0.05$.
